# A transanal alpha-loop tube facilitates endoscopic submucosal dissection of a distal rectal tumor

**DOI:** 10.1055/a-2356-7588

**Published:** 2024-07-15

**Authors:** Yuka Kagaya, Yoshikazu Hayashi, Takaaki Morikawa, Hiroki Hayashi, Hisashi Fukuda, Stefano Kayali, Hironori Yamamoto

**Affiliations:** 1 12838 Department of Medicine, Division of Gastroenterology, Jichi Medical University, Shimotsuke, Japan; 2 Department of Gastroenterology, Shin-Oyama City Hospital, Oyama, Japan

Collapsing the lumen with frequent gas aspiration maintains thick submucosal tissue and stabilizes endoscope controllability during endoscopic submucosal dissection (ESD) 1 2 . We introduced the placement of drainage tubes to drain gas and fluid 3 4 . Although we proved the usefulness of a Foley catheter as a drainage route from the rectum, its cost was not cheap, at 2000 Japanese yen (JPY). We hypothesized that an nasogastric tube with a looped tip in an alpha shape ( Fig. 1 ), at 88 JPY, would instead work as well as the catheter.

**Fig. 1:**
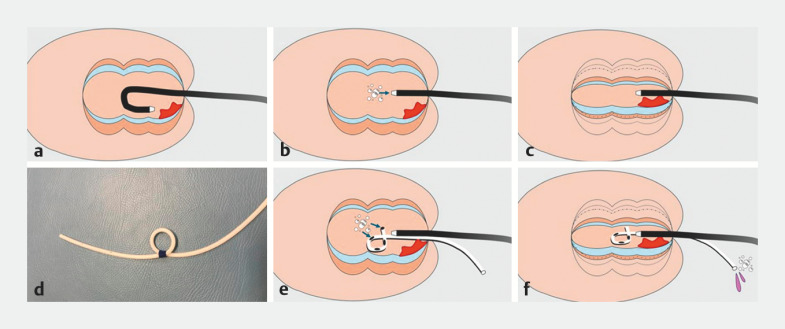
Collapsing the rectum by continuously draining gas and fluid through the nasogastric tube with alpha-loop tip during endoscopic submucosal dissection (ESD). **a** Insufflation enlarges the rectal lumen. A lesion close to the anal canal tends to be behind the rectal valve, where the submucosa is thin and endoscopic maneuvers are unstable. **b, c** Aspiration makes the rectal wall become tangential to the endoscope, the submucosa thickens, and endoscopic maneuvering is stable in the narrow space. **d** A 14-Fr nasogastric tube with an alpha-shaped looped tip is used to prevent it from withdrawing. **e, f** The alpha-loop tube placed in the rectum drains gas and fluid, including blood, spontaneously, without intervention, which results in collapse of the rectal lumen, stabilization of endoscopic maneuvers, thickening of the submucosa, and avoidance of clot accumulation. The irrigated saline washes away fluid, including blood, and drains through the catheter, facilitating ESD.

A 79-year-old woman was referred for endoscopic resection of a tumor of 3 cm in diameter in the distal rectum. During colonoscopy, the tumor was suspected of being an intramucosal tumor and biopsy was also suggestive of adenoma ( Fig. 2 ). The pocket-creation method of ESD was performed ( Video 1 ).

**Fig. 2:**
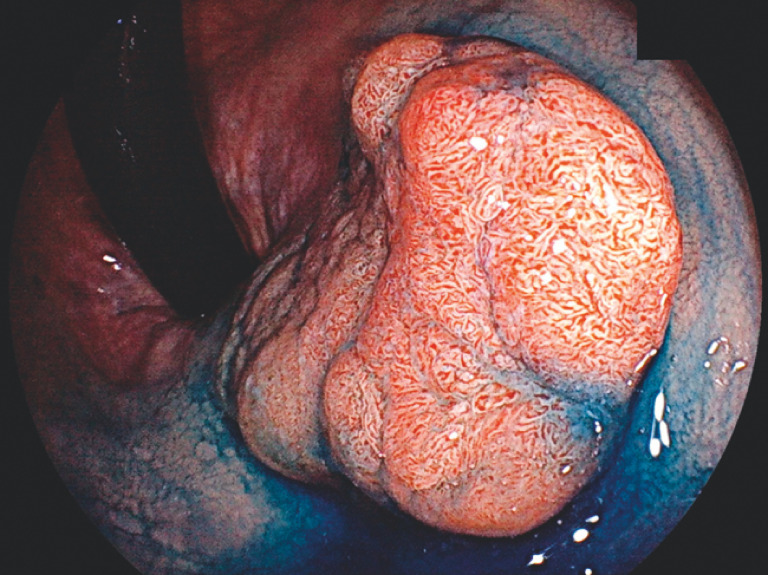
A 3-cm sessile tumor in the distal rectum dyed with indigo carmine.

A distal rectal tumor was dissected using endoscopic submucosal dissection with an alpha-loop tube drain.Video 1

A gastroscope (EG-840T; Fujifilm, Tokyo, Japan) was used with a conical cap (CAST hood; TOP corporation, Tokyo, Japan) 5 , carbon dioxide regulator (CW-200; Fujifilm), and FlushKnife BT-S (DK2620J-B15S-; Fujifilm). A 14-Fr nasogastric tube (SF-GX1420; Terumo, Tokyo, Japan), the tip of which was looped in an alpha shape ( Fig. 1 ), was introduced transanally while manually compressing the loop ( Fig. 3 ). Submucosal dissection was started on the distal side. Gas and fluid, including blood, drained spontaneously through the tube. The end of the tube was inserted into an open plastic bag to minimize flow resistance. During the procedure, neither the endoscopic maneuverability was affected, nor the tube position changed ( Fig. 4 ). The one-way current of saline irrigated through the accessory channel during the procedure maintained a clear field of vision. In addition, no clots accumulated in the rectum. Finally, the tumor was removed en bloc ( Fig. 5 ). The total amount of irrigated saline and drained fluid was 20 mL each.

**Fig. 3:**
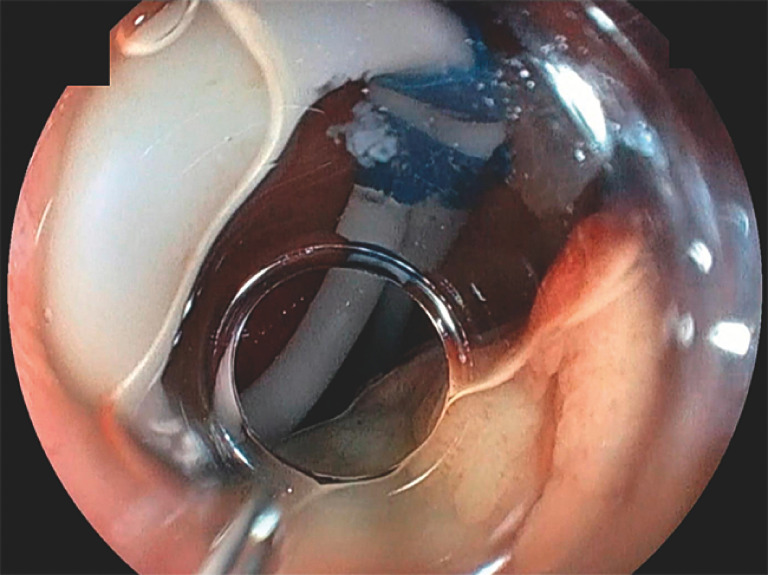
A 14-Fr nasogastric tube, the tip of which was looped in an alpha shape, was placed transanally in the rectum. The alpha-shape prevents the catheter from withdrawing.

**Fig. 4:**
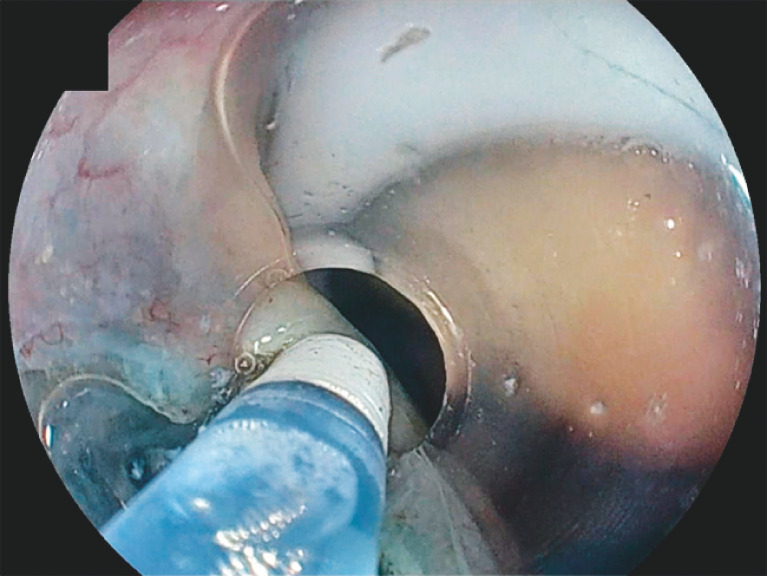
When incising the mucosa, clamping the drainage tube enabled insufflation.

**Fig. 5:**
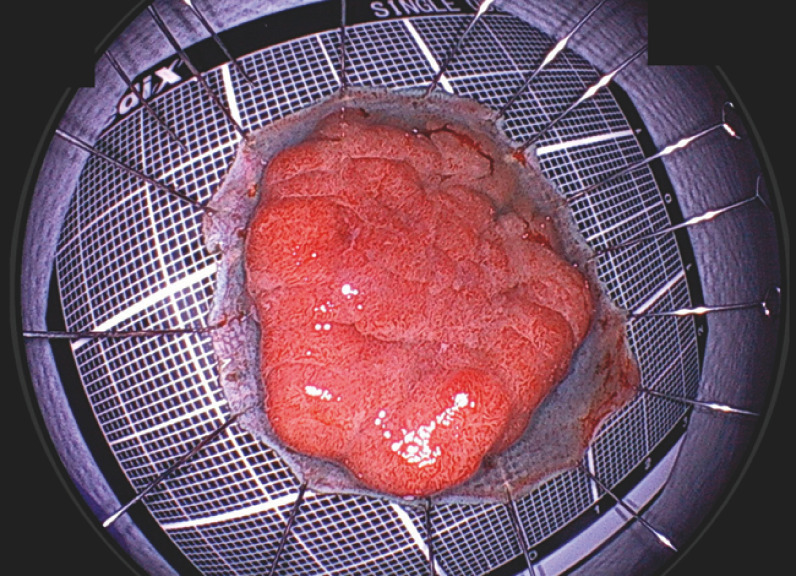
The sessile tumor was completely removed without adverse events. Pathology was low grade dysplasia with negative margins.

An alpha-loop nasogastric tube facilitated ESD of a distal rectal cancer as a cheap alternative to a Foley catheter.

Endoscopy_UCTN_Code_CCL_1AF_2AZ

## Notes

Endoscopy E-Videos https://eref.thieme.de/e-videos
*E-Videos* is an open access online section of the journal *Endoscopy* , reporting on interesting cases and new techniques in gastroenterological endoscopy. All papers include a high-quality video and are published with a Creative Commons CC-BY license. Endoscopy E-Videos qualify for HINARI discounts and waivers and eligibility is automatically checked during the submission process. We grant 100% waivers to articles whose corresponding authors are based in Group A countries and 50% waivers to those who are based in Group B countries as classified by Research4Life (see: https://www.research4life.org/access/eligibility/ ). This section has its own submission website at https://mc.manuscriptcentral.com/e-videos .
